# Decomposing Spatial β‐Diversity in the temperate forests of Northeastern China

**DOI:** 10.1002/ece3.7926

**Published:** 2021-07-22

**Authors:** Changtuan Yu, Chunyu Fan, Chunyu Zhang, Xiuhai Zhao, Klaus von Gadow

**Affiliations:** ^1^ Research Center of Forest Management Engineering of State Forestry and Grassland Administration Beijing Forestry University Beijing China; ^2^ Faculty of Forestry and Forest Ecology Georg‐August‐University Göttingen Göttingen Germany

**Keywords:** latitudinal gradient, life‐form, nestedness, turnover, β‐diversity

## Abstract

β‐Diversity, which describes the extent of change in species composition in a given region, has become a core issue in ecology in recent years. However, it is hard to understand the underlying mechanisms of β‐diversity by using indices that yield identical values under species replacement and nestedness pattern. Partitioning β‐diversity into turnover (caused by species replacement among plots) and nestedness components (caused by species loss or gain among plots) may provide improved understanding of the variation in species composition. Here, we collected presence–absence data of 456 one‐tenth ha circular plots in the temperate forests of Northeastern China spanning a latitudinal range of 12° (41–53°N). We decomposed β‐diversity to assess the relative contribution of the turnover and nestedness components across latitudinal gradients. We used regression analysis to assess the relationship between spatial distance and β‐diversity. We applied variation partitioning to evaluate the importance of the measured environmental and spatial variables in explaining β‐diversity. We used the Tukey honest significant difference test to test the differences of β‐diversity along latitudinal gradients. Pearson correlations (*r*) and significance (*p*‐value) were computed using the Mantel tests to verify the relationship between distance and β‐diversity. The ANOVA test was used to verify whether the variation of β‐diversity explained by the environment and distance was significant. Our results showed that (1) β‐diversity and the turnover component were higher at low latitudes (zones A and B) than at high latitudes (zones C and D), while there was no relationship between the nestedness component and latitude. (2) The turnover component was dominant. (3) The spatial distance explained more variation of β‐diversity than the measured environmental factors. Therefore, we conclude that β‐diversity is mainly a product of species turnover in our temperate forests, suggesting that different localities harbor different species. We find that decomposing β‐diversity into the turnover and nestedness components is a useful approach to explore the variation of community composition and their causes.

## Graphical abstract

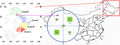

## 1 INTRODUCTION

β‐Diversity, an essential component of biodiversity, describes the change in community species composition at temporal and spatial scales. Whittaker (1960) proposed the concept of β‐diversity and defined it as the extent of change in species composition between plots within a given region. During recent decades, β‐diversity has received much attention and has become a key topic of ecological research (Anderson et al., 2011; Sutherland et al., 2013; Tan et al., 2017; Wilson & Shmida, 1984). Detecting patterns of β‐diversity is crucial for understanding ecological processes of community assembly (Kraft et al., 2011; Myers et al., 2013; Myers & LaManna, 2016) and formulating effective biodiversity conservation measures (Baselga, 2010; Gianuca et al., 2017; Gutiérrez‐Cánovas et al., 2013; Socolar et al., 2015).

Latitudinal patterns of β‐diversity are variable and remain controversial (Chen et al., 2010). For example, β‐diversity has been found to be positively, negatively, or not at all correlated with latitude (Gaston et al., 2007; Kraft et al., 2011; Paknia & Sh, 2015; Qian & Ricklefs, 2007; Soininen et al., 2007; Tang et al., 2012). Koleff et al. (2003) reviewed 15 possible relationships between β‐diversity and latitude with seven negative, two positive, and six non‐significant relationships. The variety of relationships may be caused by differences in the taxa (Hao et al., 2019). For example, Qian and Ricklefs (2007) and Tang et al. (2012) showed that plant β‐diversity decreased with increasing latitude in North America and China, respectively. Gaston et al. (2007) showed that the relationship between global avifauna β‐diversity and latitude was insignificant. Qian et al. (2005) found a negative relationship between the β‐diversity of angiosperms and latitude in temperate floras of Eastern Asia and eastern North America. The specific geographical region, the size of sampling units, and different β‐diversity metrics used in each study also resulted in the variety of latitudinal gradients of β‐diversity.

The influence of environmental variables and spatial distance on β‐diversity has been the subject of much research in recent years (De Cáceres et al., 2012; Qian, 2009; Qian & Ricklefs, 2007; Xing et al., 2019). Qian and Ricklefs (2007) explored the explanatory power of spatial distance and climate difference on β‐diversity using the list of vascular plants in North America. Zhang et al. (2020) studied the effects of 17 environmental variables and the latitude on β‐diversity in the temperate forests of Northeastern China. Tang et al. (2012) and Xing et al. (2019) reported that climate factors were crucial indicators influencing the latitudinal gradient pattern of β‐diversity. Tan et al. (2017) showed that dispersal limitation increased β‐diversity by facilitating species aggregation at small scales in the Changbai Mountains. Both environmental filtering and dispersal limitation contribute to specific patterns of β‐diversity (De Cáceres et al., 2012; Qian, 2009; Qian & Ricklefs, 2007; Xing et al., 2019). However, the relative importance of these two processes differs between temperate and tropical regions (Myers et al., 2013).

β‐Diversity can reflect two different types of variation in species composition, spatial turnover and nestedness (Harrison et al., 1992; Lennon, 2001). Spatial turnover refers to species replacement among plots due to spatial distance or environmental heterogeneity, causing differences in species composition (Koleff et al., 2003; Qian et al., 2005). Nestedness refers to the ordered loss (or gain) of species along environmental or ecological gradients (Almeida‐Neto et al., 2008; Ulrich et al., 2009). Species replacement and species loss (or gain) represent not only different but also antithetic ecological mechanisms (Baselga, 2010; Podani & Schmera, 2011). Disentangling the turnover and nestedness components may provide a unique way to understand the variation of species composition.

Recently, using methodological advances in the decomposition of β‐diversity, the total β‐diversity can be additively decomposed into turnover and nestedness components (Baselga, 2010). Baselga (2010) showed that the total β‐diversity of European longhorn beetle was similar for northern and southern Europe. However, both turnover and nestedness contributed strongly to β‐diversity in northern Europe whereas β‐diversity was mainly a product of turnover in southern Europe. It is thus possible to interpret a β‐diversity pattern incorrectly without the distinction between turnover and nestedness (Da Silva et al., 2018; Fontana et al., 2020; Gutiérrez‐Cánovas et al., 2013). Si et al. (2015) showed that the turnover component contributed dominantly to β‐diversity both for birds and for lizards in the Thousand Island Lake, China, while the nestedness component increased with differences in area. In a study that involved the β‐diversity of macrobenthos, natural factors (elevation, salinity) mainly affected the turnover of β‐diversity, while human disturbance (acidity, metals) had a greater impact on nestedness (Gutierrez‐Canovas et al., 2013). The contrasting effects of turnover and nestedness on β‐diversity patterns have significant implications for species and habitat conservation (Gutiérrez‐Cánovas et al., 2013; Si et al., 2015).

Northeastern China is home to the most extensive area of natural forests in China. Because of its high plant species richness and broad geographical range, Northeastern China is an ideal area for studying large‐scale biodiversity patterns (Luo et al., 2019; Zhang et al., 2020). In this study, the analysis was applied to the overall community and each life‐form (trees, shrubs, and herbs). Based on field survey data of temperate forests in Northeastern China, this study attempts to answer three questions: (1) Which component (turnover and nestedness) of β‐diversity is dominant, or do both components contribute similarly to β‐diversity? (2) Does β‐diversity decrease or increase with increasing latitude? And (3) which explanatory variables (measured environmental factors, spatial distance) contribute more to β‐diversity?

## 2 MATERIALS AND METHODS

### 2.1 Study area

The study area in Northeastern China covers seven important mountain ranges: the Greater and Lesser Khingan (i.e., DXA and XXA), the Wanda (WDS), the Zhangguangcailing (ZGC), the Laoyeling (LYL), the Hadaling (HDL), the Changbai (CBS), and the Longgang (LGS) Mountain areas (Zhang et al., 2020). The latitudinal range of the study region spans 12° degrees, from 41 to 53°N, including three climatic zones: warm temperate zone, temperate zone, and cold temperate zone. Rainfall is mainly concentrated during the months of July to September. The annual rainfall varies from 474.46 to 813.47 mm and decreases with increasing latitude. The average annual temperature ranges from −2.93 to 6.81°C, showing a downward trend with increasing latitude.

The main vegetation types in the study region are warm temperate deciduous broad‐leaved forests, temperate coniferous broad‐leaved mixed forests, and cold temperate *Larix gmelinii* forests. The main tree species are *Quercus acutissima*, *Pinus koraiensis*, *Quercus mongolica*, *Tilia amurensis*, *Abies fabri*, *Larix gmelinii*, *Betula platyphylla*; the main shrub species are *Lespedeza bicolor*, *Corylus mandshurica*, *Lonicera japonica*, *Salix hsinganica*, *Ledum palustre*, and *Pinus pumila*; and among the most common herb species are *Carex rigescens*, *Poa annua*, *Sanguisorba officinalis*, and *Carex callitrichos*.

### 2.2 Forest plot network and data acquisition

#### 2.2.1 Plot layout

**FIGURE 1:**
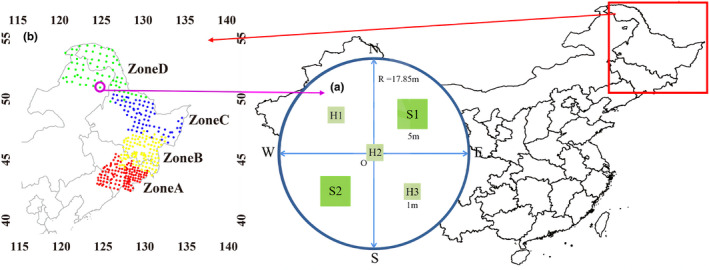
Location of the studied forest plots and design of the plot layout in Northeastern China. (a) Design of the plot layout. For each 0.1‐ha circular plot, two 5 m × 5 m quadrats (indicated by the symbol S1 and S2) were selected to study shrubs. Three 1 m × 1 m quadrats (indicated by the symbol H1, H2, and H3) were used to study herbs. (b) Map showing the division of the forest plots into four latitudinal zones. To make the sizes of the latitudinal zones more equal (to the best of our ability) and simultaneously taking common plant associations into account, we divided the whole study area into four latitudinal zones A, B, C, and D from the lowest to the highest latitude. Zones A and B and zones B and C are further subdivided according to mountain systems. Zone D includes the Greater Khingan and the upper parts of the Lesser Khingan because the vegetation type of the upper range of the Lesser Khingan is similar to the Greater Khingan

In the summer of 2017 and 2018, a total of 456 circular field plots were established following similar studies on plant diversity (Fang et al., 2012; Stohlgren et al., 1995; Wu et al., 2019; Zhang et al., 2020). The network of 0.1‐ha sample plots covers the whole study region. In each plot, two 5 m × 5 m quadrats (indicated by the symbol S1 and S2) were selected to study shrubs. Three 1 m × 1 m quadrats (indicated by the symbol H1, H2, and H3) were used to study herbs (Figure 1a).

#### 2.2.2 Data acquisition

For each 0.1‐ha circular plot, all trees with DBH ≥5 cm and shrubs in two 5 m × 5 m subplots with DBHs <5 cm were measured and stem‐mapped. Herbs were surveyed in three 1 m × 1 m subplots of each 0.1‐ha circular plot. All plants used in this study were identified at species level.

The environmental variables include the soil depth, altitude, litter thickness, average annual temperature, temperature difference, and annual rainfall. The soil depth, altitude, and litter thickness were measured and recorded in the field. The average annual temperature, temperature difference, and annual rainfall were collated from the global meteorological data website (WorldClim database V.1; vide Hijmans et al., 2005). The longitude and latitude of the sample plots used to calculate distance between plots (treated as distance variable) were measured using a GPS device.

### 2.3 Data analysis

#### 2.3.1 Latitudinal zones division and Sampling completeness estimate

We divided the whole study area into four latitudinal zones A, B, C, and D from the lowest to the highest latitude. Zones A and B and zones B and C were further subdivided according to mountain boundary. Zone D includes the Greater Khingan and the upper parts of the Lesser Khingan because the vegetation type of the upper range of the Lesser Khingan was similar to the Greater Khingan (Figure 1b). β‐Diversity measures the extent of species composition variation among communities. The local contribution to beta diversity (LCBD) was found to be significantly correlated with species composition in several studies (e.g., Legendre & De Cáceres, 2013; Qiao et al., 2015; Hill et al., 2021). We calculated LCBD as an auxiliary variable which supports our latitudinal zone division. The results presented in the Supporting Information (Appendix S1) showed that there was a significant difference (*p*‐value < 0.001) between each possible pair of zones except for three pairs in the high latitude, which suggested that the binning of latitudinal zones was reasonable and acceptable.

We verified our sampling completeness by estimating sample coverage using the function “iNEXT” in the R (https://www.r‐project.org/) package “iNEXT” (Chao et al., 2020). The result presented in the Supporting Information (Appendix S2) showed that sample coverage of the overall community and each kind of life‐form all reached a steady state before the number of sample plots was maximized. This observation indicated that the sample coverage was sufficient in our latitudinal zones.

#### 2.3.2 β‐Diversity decomposition

Using the decomposition method of β‐diversity proposed by Baselga (2010), the β‐diversity was divided into turnover and nestedness components based on pairwise‐site comparisons (Baselga, 2010). The significant differences of β‐diversity and decomposition components (turnover and nestedness) among latitudinal zones were tested using the Tukey honest significant difference test (the function “TukeyHSD” in R software). The specific decomposition method including the meaning of symbols (*a*, *b*, and *c*, i.e., the components of β‐diversity for presence–absence data) was described in detail in Baselga (2010).

#### 2.3.3 Regression analysis and variation partitioning

We regressed the pairwise‐site β‐diversity on the spatial distance between plots to test how β‐diversity changes with the spatial distance. The distance variable was transformed from latitude and longitude of each plot using function “distm” in the R package “geosphere”. The significance of the Pearson correlations was computed based on Mantel permutation tests using the R package “vegan”.

The environmental variables include the soil depth, altitude, litter thickness, average annual temperature, temperature difference, and annual rainfall. Spatial distance variable was constructed using the function “dbMEM” in the R package “adespatial”. We used the function “forward.sel” in the R package “adespatial” to select the significant spatial and environmental variables. These significant spatial and environmental variables were used to partition the variation in the β‐diversity into individual components accounted for by measured environmental variables and spatial distance variables. Variation partitioning was carried out using the function “varpart” in the R package “vegan”. An ANOVA test was used to verify whether the variation explained by the environment and distance was significant.

## 3 RESULTS

### 3.1 Species diversity

The entire study region has 492 species (53 trees, 97 shrubs, and 342 herbs species), belonging to 85 families and 279 genera (Appendix S3). The number of species, genera, and families of the three life‐forms (tree, shrub, and herb) decreased monotonously from low to high latitude. Some species were distributed widely, occurring throughout the entire study region, whereas some species only occurred within a specific latitudinal zone. For example, *Betula platyphylla*, *Carex callitrichos*, *and Cardamine leucantha* were found in the whole study region; *Syringa reticulata* was found in three latitudinal zones but not in zone D; *Acer barbinerve*, and *Acer pictum* were widely distributed in zones A and B, while *Larix gmelinii*, *Pinus pumila*, and *Ledum palustre* were mostly found in zone D.

### 3.2 Latitudinal gradients and decomposition components of β‐diversity

**FIGURE 2:**
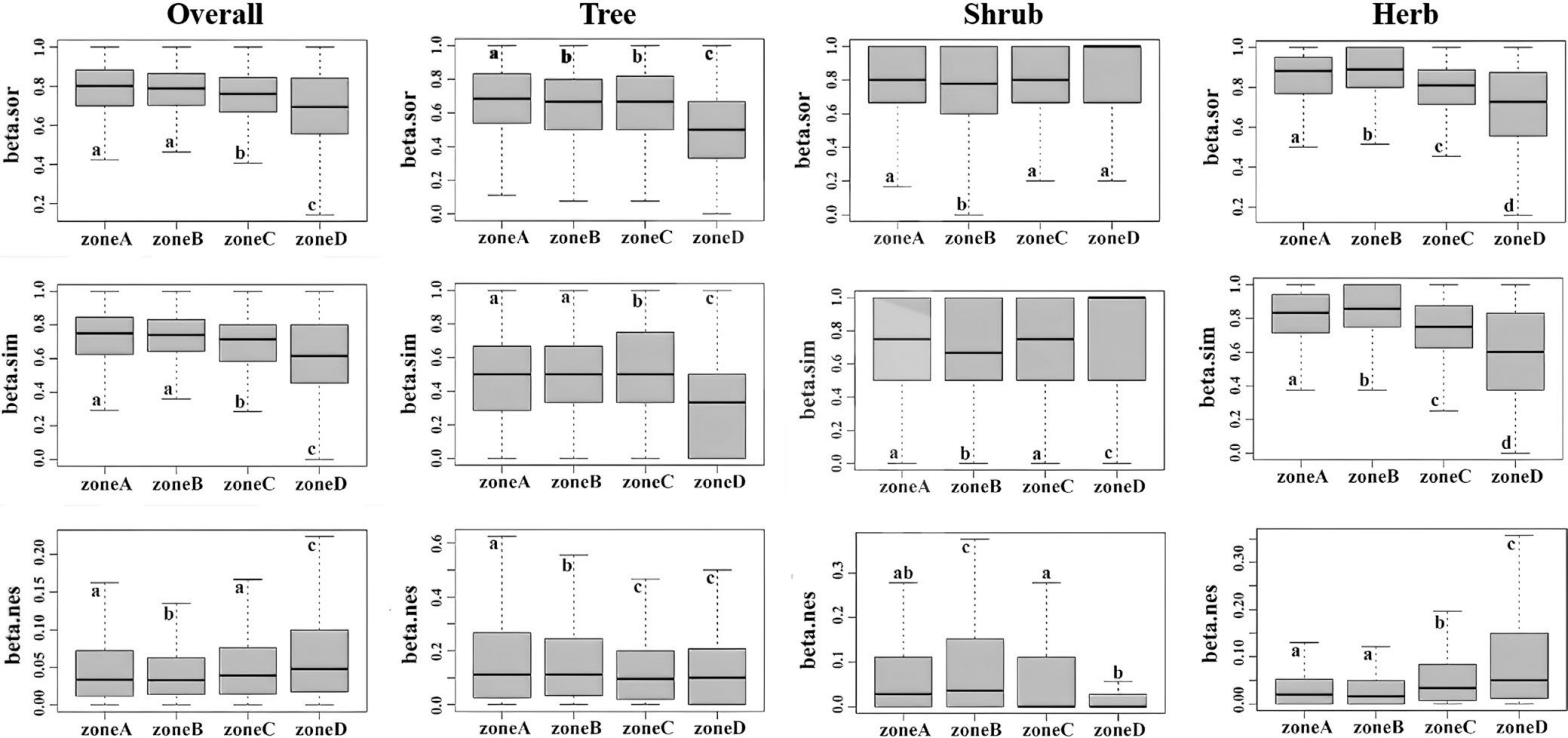
β‐Diversity (beta.sor) and its decomposition components (beta.sim and beta.nes) of the overall community and each kind of life‐form on latitudinal gradients. Columns from left to right represent β‐diversity and its decomposition components for the overall community (indicated by Overall) and three life‐form of plants (indicated by Tree, Shrub, and Herb, respectively). Different lowercase letters indicate a significant difference (*p*‐value < 0.05) between them

The results show that, in terms of the relative contribution of the turnover and nestedness component to β‐diversity, the turnover component was dominant both for the overall community and for each life‐form (Figure 2). The total β‐diversity and the turnover component of the overall community and each life‐form, except for shrubs, were greater at low latitudes than at high latitudes. The latitudinal gradients of β‐diversity were not steep, but differences were nevertheless significant (Figure 2). Both total β‐diversity and two decomposition components of the shrubs did not show a significant latitudinal gradient. There was no relationship between the nestedness component of both the overall community and each life‐form with latitude (Figure 2).

### 3.3 The relationship between β‐diversity, spatial distance, and environmental factors

#### 3.3.1 The relationship between β‐diversity and spatial distance

**FIGURE 3:**
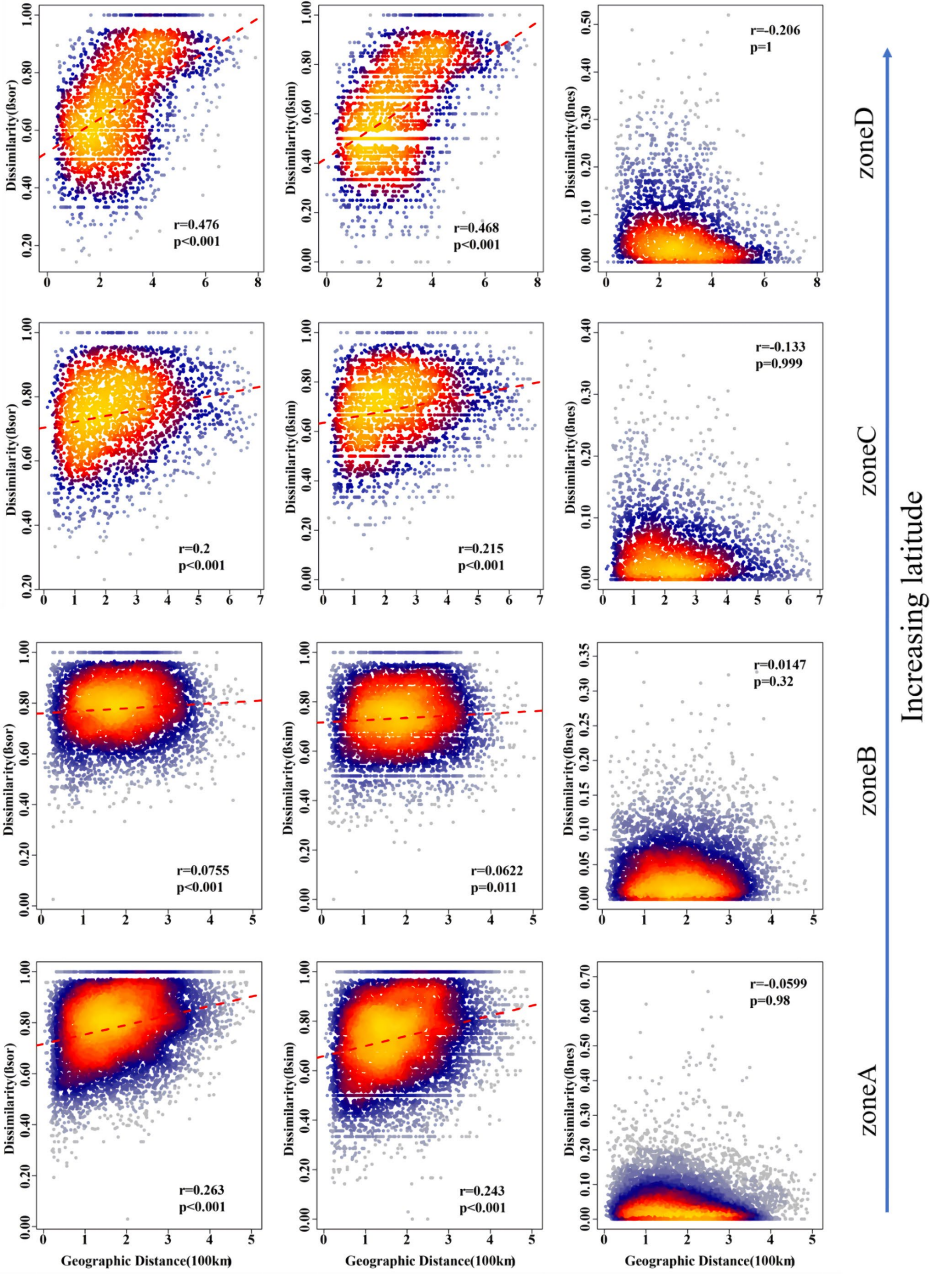
The relationships between geographical distances and β‐diversity (βsor) and its decomposition components (βsim and βnes) of the overall community. Pearson correlation (*r*) and significance (*p*‐value, computed using Mantel tests) are shown. In this figure, different colors represent the frequency of the pairwise‐site values of β‐diversity. It was greatest for deep orange, followed by red, blue, and smallest for gray

Both the total β‐diversity and the turnover component of the overall community increased with increasing distance between plots (Figure 3). However, the nestedness component of β‐diversity was not correlated with the spatial distance. In terms of each kind of life‐form, the Mantel test showed that the β‐diversity and its turnover components were significantly correlated with spatial distance (Appendix S4), except for zone B of trees (*p*‐value = 0.136 and 0.406). The nestedness components of β‐diversity showed no significant relationship with the distance between sample plots (Appendix S4).

#### 3.3.2 The relative importance of spatial distance and measured environmental variables

**FIGURE 4:**
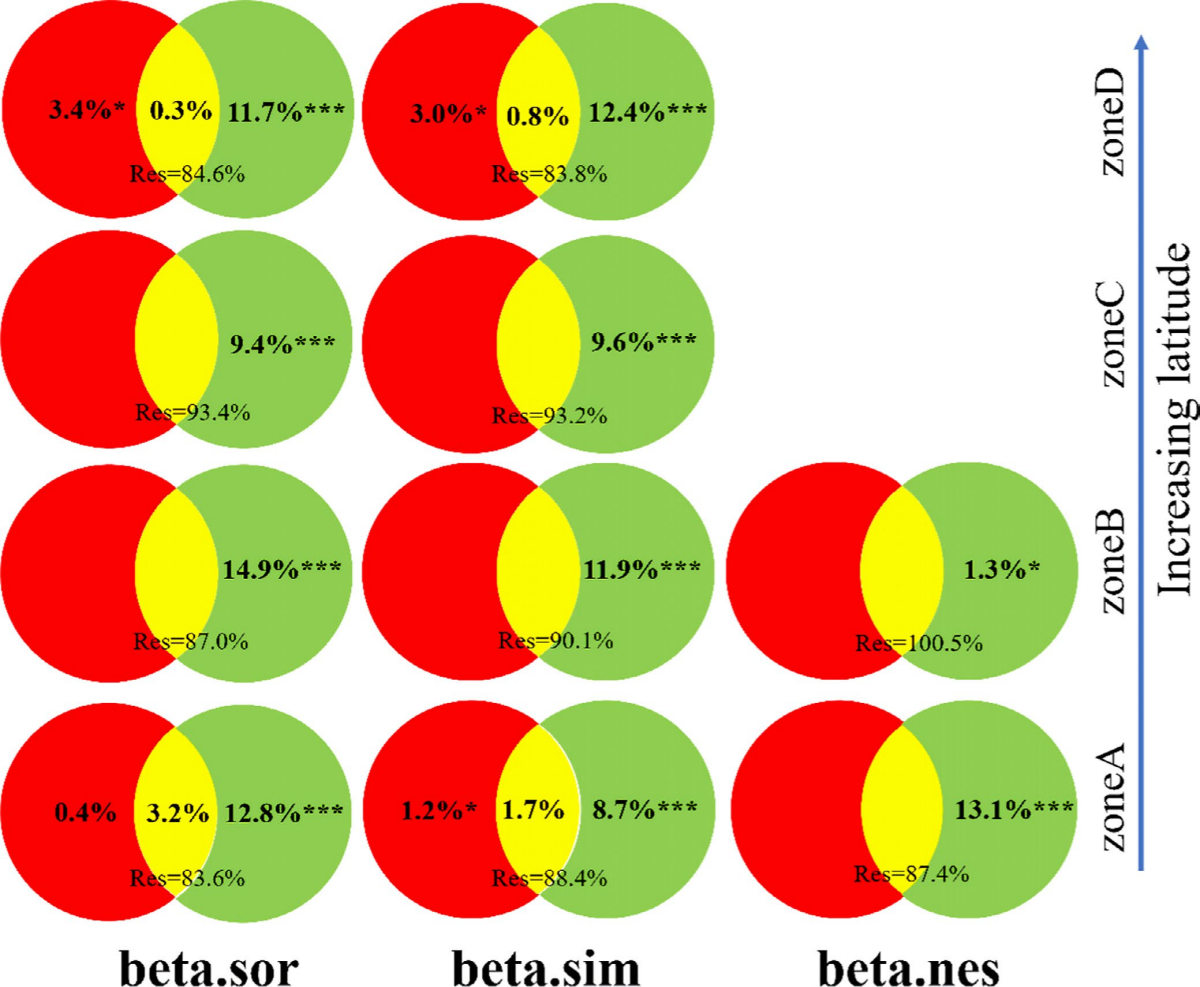
Venn diagrams showing the variation partitioning of the change in total β‐diversity (beta.sor), the turnover (beta.sim), and nestedness (beta.nes) component within each of four latitudinal zones (A to D). The numbers in the diagrams are percentage (i.e., %, values ≤0 not shown), which are used to estimate the proportion of variation explained by spatial distance (left circle, red color) and measured environmental variables (right circle, green color). The sums of the proportions of Spatial, Environmental, and Residual do not always exactly add to 1. If the sum is less than 1, there was another proportion explained by the combination of Spatial and Environmental. If the sum is greater than 1, the proportion explained by the explanatory variables (Spatial, Environmental, or the combination of Spatial and Environmental) was negative. A negative value indicates that the proportion explained by the explanatory variables was less than the random value (Borcard et al., 2019; Legendre & Legendre., 2012). *represents *p*‐value < 0.05, **represents *p*‐value < 0.01, ***represents *p*‐value < 0.001, and Res represents residuals. The missing position in the diagrams indicates that there are neither significant environmental variables nor significant spatial variables at this location, so a variation partitioning cannot be performed

The spatial distance explained 12.8%, 14.9%, 9.4%, and 11.7% of the variation of total β‐diversity of the overall community from zones A to D (Figure 4). In terms of the turnover component, the proportion explained by the spatial distance from zones A to D were 8.7%, 11.9%, 9.6%, and 12.4%, respectively (Figure 4). However, for the nestedness component, the proportion explained by the spatial distance was only significant at zones A and B, where the explanatory power was 13.1% and 1.3%, respectively (Figure 4). Compared with the spatial distance, the explanatory power of measured environmental variables to β‐diversity and its decomposition components (turnover and nestedness) were relatively weak. The explanatory power was less than 5% in zones A and D (Figure 4).

The variation partitioning results of β‐diversity of each kind of life‐form showed that the measured environmental variables explained the variations of β‐diversity only in specific cases (e.g., total β‐diversity of zone A for trees, and total β‐diversity and the turnover component of zones A and D for herbs, see Appendix S5). Except for the shrubs in zone C, the spatial distance remains explanatory at all four latitudinal zones while its explanatory power was more significant than that of the measured environmental variables (Appendix S5).

## 4 DISCUSSION

In this study, we evaluated the latitudinal gradients of β‐diversity, explored which component (turnover and nestedness) of β‐diversity is dominant and assessed the relative importance of the spatial distance and measured environmental variables to β‐diversity using the presence–absence data collected from the temperate forests in Northeastern China. We used pairwise‐site comparisons (following Baselga, 2010) to calculate total β‐diversity and decomposed total β‐diversity into turnover and nestedness components. We applied regression analysis to assess the trend of β‐diversity with spatial distance (Nekola & White, 1999; Qian & Ricklefs, 2007). We conducted variation partitioning (Borcard et al., 2019; Legendre & Legendre., 2012) to analyze the relative importance of the measured environmental factors and spatial distance on β‐diversity. The analysis involved the entire community and each life‐form (trees, shrubs, and herbs). In general, our study revealed that (1) β‐diversity at relatively low latitudes (zones A and B) was greater than at high latitudes (zones C and D) in the temperate forests in Northeastern China. (2) β‐Diversity was mainly a product of species turnover. (3) Spatial distance contributed more to β‐diversity than measured environmental factors.

### 4.1 The latitudinal gradient pattern of β‐diversity and its decomposition components

The results show that total β‐diversity of the entire community and each life‐form was greater at low latitudes (zones A and B) than at high latitudes (zones C and D). The latitudinal gradients of β‐diversity are not steep, but the differences are nevertheless significant. This result is similar to the results of several previous studies (De Cáceres et al., 2012; Kraft et al., 2011; Qian, 2009; Qian & Ricklefs, 2007; Tang et al., 2012). Some studies investigated the latitudinal pattern of β‐diversity based on a plant species distribution map (Koleff et al., 2003; Qian, 2009; Qian & Ricklefs, 2007), while others used systematic inventory data of forest communities (Tang et al., 2012; Zhang et al., 2020). Some studies used species composition dissimilarity indices, that is, Sørensen, Jaccard, and Simpson, to quantify β‐diversity (Qian & Ricklefs, 2007; Tang et al., 2012). Others used the slope of the species–area relationships and the decaying rate of similarity in species composition (Drakare et al., 2006; Qiao et al., 2012). Although the forms of data and the methods used in these studies are different, they all agree that β‐diversity is greater at low latitudes than at high latitudes, which shows that the latitudinal pattern of large‐scale β‐diversity is robust (Qian & Ricklefs, 2007; Tang et al., 2012).

We divided β‐diversity into turnover and nestedness components and explored the trend with latitudinal gradient. Our results showed that both total β‐diversity and the turnover components were greater at the low latitudes (zones A and B) than at high latitudes (zones C and D). As mentioned before, the differences of β‐diversity between high latitudes and low latitudes are significant although the latitudinal gradients are not steep. Which indicated greater differences between community species composition at low latitudes than at high latitudes. In terms of the nestedness component, however, the trend corresponding to the latitudinal gradient was inconsistent. In general, we found that the latitudinal patterns of turnover and nestedness are different.

### 4.2 The relative importance of the turnover and nestedness components

In our study, the contribution of the turnover components to β‐diversity was dominant, while the contribution of the nestedness components was small. The results were similar for the different life‐forms of plants. Previous studies have also shown that the contribution of turnover components to β‐diversity is greater than that of the nestedness components under natural conditions (e.g., Gutiérrez‐Cánovas et al., 2013). The nestedness components appear to be more evident in habitats disturbed by human activities or habitats with considerable differences in species richness, such as island habitats (Gutiérrez‐Cánovas et al., 2013; Leprieur et al., 2011; Si et al., 2015). On the one hand, our study area is home to the most extensive region of natural forests in China that is almost free of human disturbance, which may be one reason for the spatial turnover dominance in β‐diversity. On the other hand, the poor connectivity among mountain systems in our study area may be another reason. Wen et al. (2016) has shown that geographical isolation is expected to be an important process in shaping the turnover patterns because the isolated mountain ranges and valleys often favor allopatric speciation (Qian et al., 2013). The contribution of the nestedness components to β‐diversity of the entire community and each plant life‐form was small. However, it seems different in each life‐form based on visual inspection. Si et al. (2015) have pointed out that the proportion of the nestedness component becomes more discernible in island habitats such as in the Thousand Islands Lake, China.

### 4.3 Ecological driving mechanism of β‐diversity and its decomposition components

Environmental filtering and dispersal limitation have been regarded as two major mechanisms of community assembly, but their relative roles across forest regions remain elusive. Our study advances previous work on assessing the importance of environmental and spatial distance in explaining β‐diversity (e.g., Morlan et al., 2008; Myers et al., 2013; Zhang et al., 2020) by decomposing β‐diversity into a turnover and a nestedness component. We found that the contribution of spatial distance to the β‐diversity and turnover components of the overall community and each kind of life‐form was more significant than that of the measured environmental variables in our temperate forests of Northeastern China. Our results are consistent with those of Morlan et al. (2008) in tropical forests and those of Wang et al. 2011, 2018 in temperate forests, but contrary to those of several previous studies (Hubbell, 2001; Myers et al., 2013). In our study, β‐diversity is almost entirely explained by spatial distance. Many studies have confirmed that dispersal limitation is significantly related to β‐diversity. For example, Qian and Ricklefs (2007) could show that geographic distance explained a large proportion of the variation in β‐diversity; nearly all of the variation in β‐diversity was attributable to geographic distance in north of 50°N. Qian (2009) showed that β‐diversity is negatively related to dispersal ability. Our study area in Northeast China is mountainous. The region is lacking connectivity, which impedes the spread of plant species, resulting in a substantial variation of species compositions among communities beyond a certain geographic distance.

Although previous work confirming the effect of environmental filtering on β‐diversity cannot be ignored (e.g., Qian & Ricklefs, 2007; Tang et al., 2012; Zhang et al., 2020), our study found that the contribution of the measured environmental variables was minimal. We speculate that the following reasons may lead to an underestimate of the role of environmental filtering. First, due to unknown historical events (e.g., ice cover, speciation, forest fires), not all plants now occupy all suitable habitats. Second, the positive and negative effects of certain environmental factors on β‐diversity may offset each other. Third, some unmeasured environmental factors may have contributed to explaining β‐diversity (Jones et al., 2008; Legendre et al., 2009). Finally, a specific environmental variable may have a particular impact on β‐diversity, but it is very difficult to take all possible environmental variables into account. This issue needs to be addressed in forthcoming studies.

## 5 CONCLUSION

Using presence–absence data of community species composition in the temperate forests in Northeastern China, we evaluated the latitudinal gradients of β‐diversity, explored which component (turnover and nestedness) of β‐diversity is dominant, and assessed the relative importance of the spatial distance and measured environmental variables to β‐diversity. Our results show that (1) total β‐diversity and the turnover component are greater at low latitudes than at higher latitudes, a result which indicates that the ratio of species replacement among plots is greater at low latitudes than at high latitudes. (2) β‐Diversity was mainly a product of species turnover in our study area, suggesting that different localities harbor different species; thus, conservation strategies aiming to maintain biodiversity need to protect sufficient localities. (3) The contribution of spatial distance to β‐diversity is found to be greater in our temperate forests than the contribution of the measured environmental variables.

## CONFLICT OF INTEREST

The authors declare that they have no competing interests.

## AUTHOR CONTRIBUTIONS

**Changtuan Yu:** Formal analysis (lead); Methodology (lead); Writing‐original draft (lead). **Chunyu Fan:** Formal analysis (supporting); Methodology (supporting); Supervision (lead). **Chunyu Zhang:** Data curation (lead); Funding acquisition (equal); Supervision (lead). **Xiuhai Zhao:** Data curation (lead); Funding acquisition (lead); Investigation (lead). **Klaus von Gadow:** Writing‐original draft (supporting).

## Supporting information

Appendix S1–S5

## Data Availability

The data that support the findings of this study can be accessed on Figshare: https://doi.org/10.6084/m9.figshare.14900583.
