# Supplementary material for: Decomposing Spatial β‐Diversity in the temperate forests of Northeastern China
Source: Ecol Evol. 2021 Jul 22;11(16):11362–72. doi: 10.1002/ece3.7926 (PMC8366879; doi:10.1002/ece3.7926)
Supplement: Supplementary file 1 — Appendix S1–S5 [file ECE3-11-11362-s001.docx]

# Supporting Information


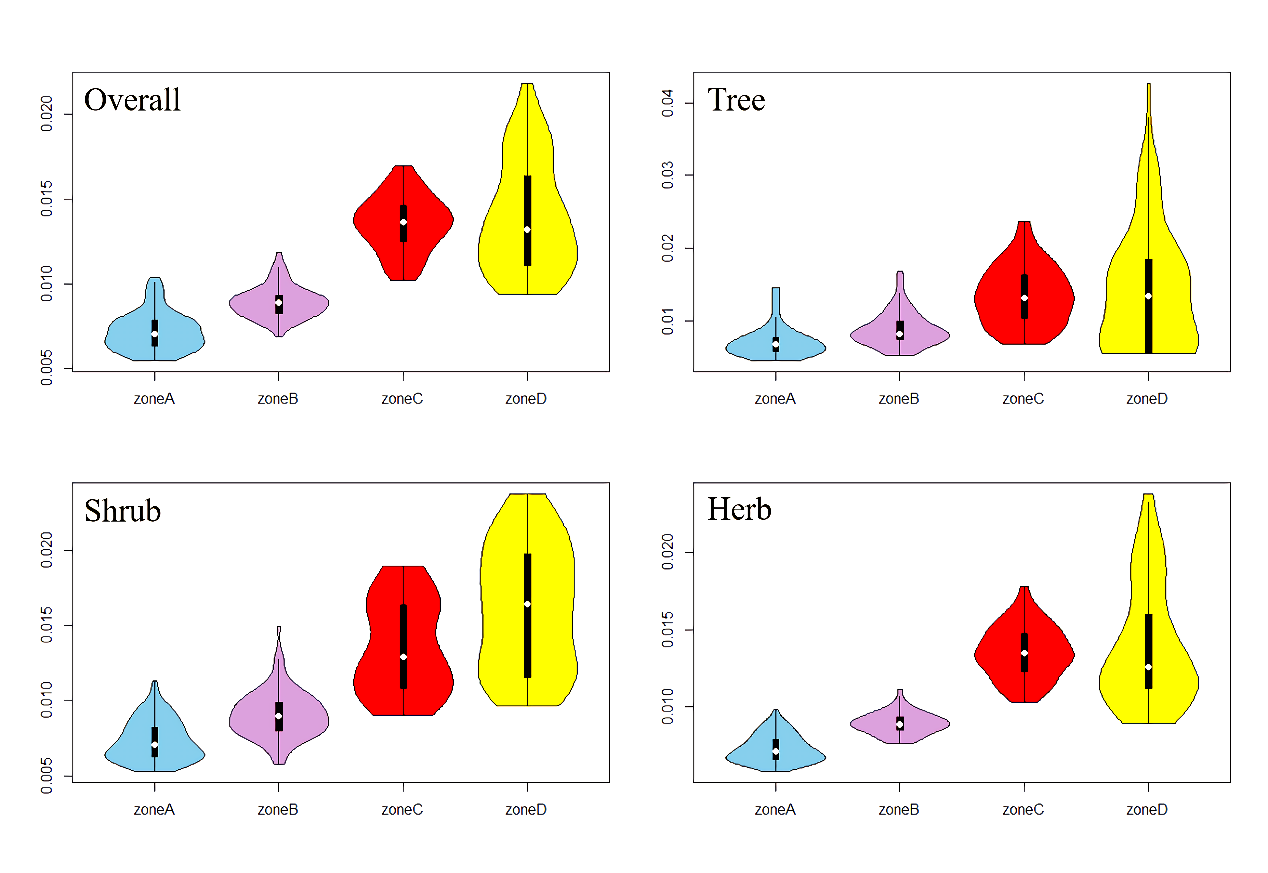


**Appendix S1.1**. The distribution of Local Contribution to Beta Diversity (LCBD) (Legendre & De Cáceres, 2013) of the overall community, trees, shrubs and herbs. The white dot indicates the median. The length and width of each violin plot denote the range of LCBD value and the frequency of a specific LCBD value of this zone, respectively. The mean LCBD values of each possible pairs of zones were tested using the Welch Two Sample t-test, the results are shown in Appendix S1.2.

Legendre, P., & De Cáceres, M. (2013). Beta diversity as the variance of community data: Dissimilarity coefficients and partitioning. *Ecology Letters, 16*(8), 951–963.

| **Appendix S1.2**. p-value of Welch Two Sample t-test | | | | |
| --- | --- | --- | --- | --- |
| zones | overall | tree | shrub | herb |
| A and B | <0.001 | <0.001 | <0.001 | <0.001 |
| A and C | <0.001 | <0.001 | <0.001 | <0.001 |
| A and D | <0.001 | <0.001 | <0.001 | <0.001 |
| B and C | <0.001 | <0.001 | <0.001 | <0.001 |
| B and D | <0.001 | <0.001 | <0.001 | <0.001 |
| C and D | 0.3824 | 0.7319 | <0.001 | 0.4475 |


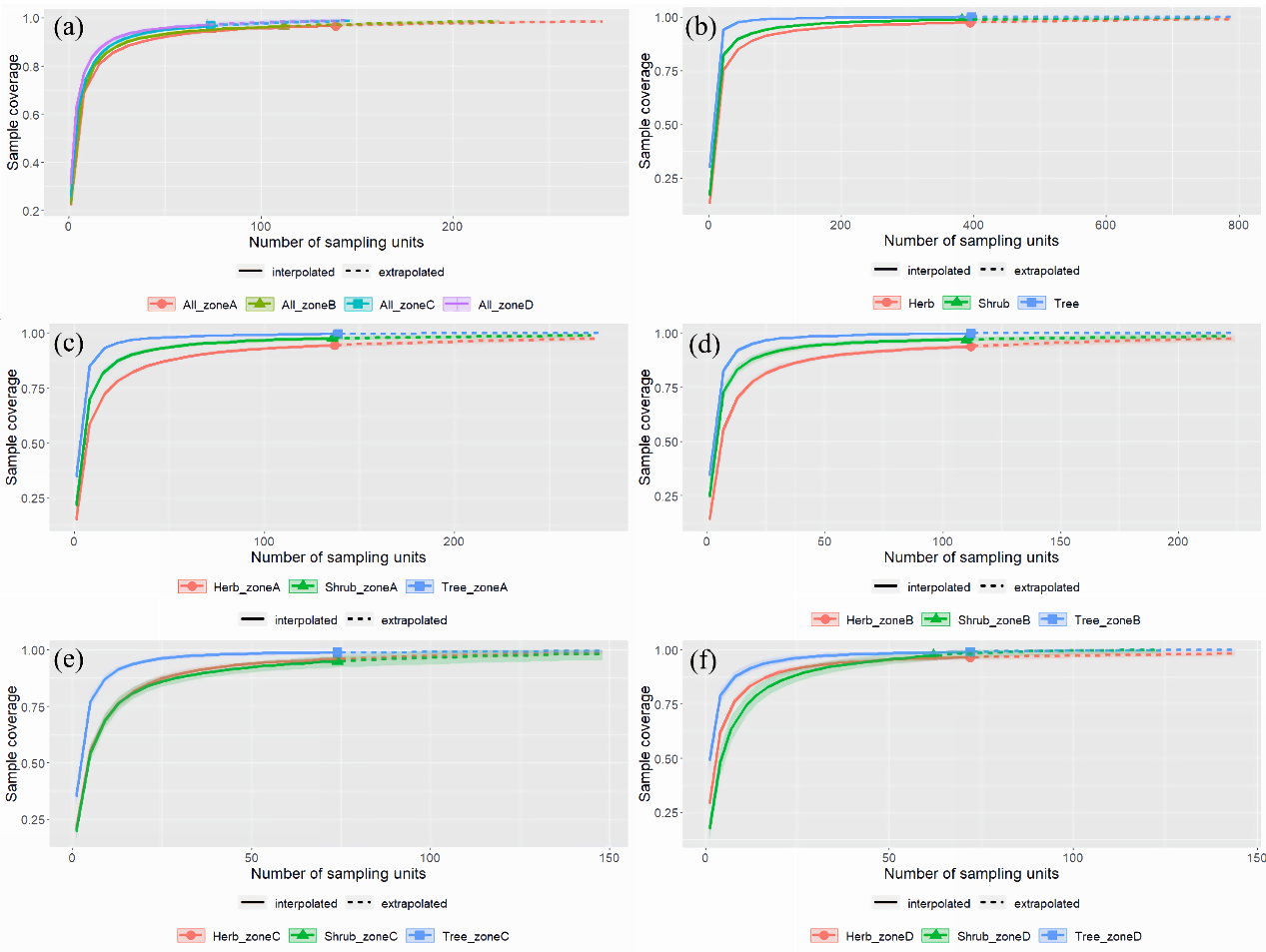


**Appendix S2**. Size-based rarefaction (solid lines) and extrapolation (dashed lines) curves up to double the reference sample size (Chao et al., 2020) for (a) the overall community (combined tree, shrub and herb species) of zone A to D, (b) the three life forms (tree, shrub and herb) of the whole study area (combined zone A ~ D), (c) each kind of life form (tree, shrub and herb) of zone A, (d) each kind of life form of zone B, (e) each kind of life form of zone C and (f) each kind of life form of zone D. Solid symbols (dots, triangles and square) denote observed data points. All shaded areas in (a)-(f) denote 95% confidence bands obtained from a bootstrap method with 100 replications. Some bands are invisible due to narrow widths.

Chao, A., Kubota, Y., Zelený, D., Chiu, C. H., Li, C. F., Kusumoto, B., ... & Colwell, R. K. (2020). Quantifying sample completeness and comparing diversities among assemblages. *Ecological Research, 35*(2), 292-314.

| Appendix S3. Species diversity in each of four latitudinal zone | | | | | |
| --- | --- | --- | --- | --- | --- |
| Latitudinal zone | Life form | Number of plots | Number of species | Number of Family | Number of Genus |
| A (HDL, CBS, LGS) | Tree | 139 | 49 | 13 | 24 |
|  | Shrub | 136 | 69 | 20 | 40 |
|  | Herb | 137 | 251 | 61 | 170 |
| B (ZGC, LYL) | Tree | 112 | 39 | 12 | 22 |
|  | Shrub | 110 | 49 | 18 | 33 |
|  | Herb | 112 | 184 | 53 | 124 |
| C (WDS, XXA) | Tree | 74 | 36 | 12 | 22 |
|  | Shrub | 74 | 49 | 17 | 30 |
|  | Herb | 74 | 110 | 38 | 84 |
| D (DXA, XXA) | Tree | 72 | 13 | 6 | 10 |
|  | Shrub | 62 | 24 | 9 | 17 |
|  | Herb | 72 | 81 | 28 | 65 |
| **Notes:** HDL, CBS, LGS, ZGC, LYL, WDS, XXA and DXA indicate the Hadaling, the Changbai, the Longgang, the Zhangguangcailing, the Laoyeling, the Wanda and the Greater and Lesser Khingan, respectively. The number of shrub and herb plots refer to independent combinations of 2 (for shrub) and 3 (for herb) subplots of each 0.1 ha circle tree plot. There are some ‘missing plots’ of shrub and herb because we did not find any shrub or herb species in 2 (shrub) or 3 (herb) subplots. | | | | | |

| **Appendix S4**. The results of the Mantel test of β-diversity (beta) and its decomposition components (turnover and nestedness) of tree, shrub and herb with spatial distance between plots. | | | | | | | | | | |
| --- | --- | --- | --- | --- | --- | --- | --- | --- | --- | --- |
| Zone | Mantel test | Tree | | | Shrub | | | Herb | | |
|  |  | beta | turnover | nestedness | beta | turnover | nestedness | beta | turnover | nestedness |
| A | r | 0.122^**^ | 0.067^*^ | 0.009 | 0.193^***^ | 0.154^***^ | 0.043 | 0.245^***^ | 0.24^***^ | -0.136 |
| B | r | 0.027 | 0.007 | 0.02 | 0.056^**^ | 0.043^*^ | -0.011 | 0.135^***^ | 0.106^***^ | 0.005 |
| C | r | 0.158^***^ | 0.149^**^ | 0.057 | 0.132^***^ | 0.149^**^ | -0.123 | 0.18^***^ | 0.194^***^ | -0.132 |
| D | r | 0.32^***^ | 0.331^***^ | -0.158 | 0.304^***^ | 0.291^***^ | -0.164 | 0.426^***^ | 0.395^***^ | -0.171 |
| **Notes：*** represent *p-value* < 0.05，** represent *p-value* < 0.01 and *** represent *p-value* < 0.001. | | | | | | | | | | |

| **Appendix S5**. The proportion of total β-diversity (beta) and its decomposition components (turnover and nestedness) of each kind of life form explained by environmental factors and spatial distance in four latitudinal zones. | | | | | | | | | | |
| --- | --- | --- | --- | --- | --- | --- | --- | --- | --- | --- |
| zone | Variables | Trees | | | Shrubs | | | Herbs | | |
|  |  | beta | turnover | nestedness | beta | turnover | nestedness | beta | turnover | nestedness |
| A | Spatial | 11.5^***^ | 6.6^**^ | 17.9^***^ | 14.2^***^ | 11.3^***^ | 3.5^***^ | 15.3^***^ | 15.3^***^ | 11.6^***^ |
|  | Environmental | 2.6^***^ | — | — | 1.7^**^ | 1.1^*^ | 0.6^*^ | 1.2^*^ | 1.5^*^ | — |
|  | Residual | 85.8 | 94.9 | 83.6 | 83.4 | 85.6 | 95.6 | 80.3 | 80.1 | 89.9 |
| B | Spatial | 14.4^***^ | 4.8^**^ | 10.1^***^ | 7.4^***^ | 10.4^***^ | 5.8^***^ | 8.1^***^ | 7.2^***^ | — |
|  | Environmental | — | — | — | — | — | — | — | — | — |
|  | Residual | 87.5 | 97 | 91.8 | 94.5 | 91.5 | 96.1 | 93.8 | 94.7 | — |
| C | Spatial | 18.5^***^ | 8.7^**^ | 2.3^**^ | — | — | — | 10.1^***^ | 10.4^***^ | 3.9^**^ |
|  | Environmental | — | — | — | — | — | — | — | — | — |
|  | Residual | 84.3 | 94.1 | 100.5 | — | — | — | 92.7 | 92.5 | 98.9 |
| D | Spatial | 5.7^**^ | 15.2^***^ | — | 13.6^***^ | 7.2^**^ | — | 12.5^***^ | 10.6^***^ | — |
|  | Environmental | — | — | — | — | — | — | 3.1^**^ | 1.3^.^ | — |
|  | Residual | 97.2 | 87.7 | — | 89.8 | 96.2 | — | 84.2 | 86.8 | — |
| **Notes：**The numbers in the table are percentage (i.e. %, values≤0 not shown). * represent *p-value* < 0.05，** represent *p-value* < 0.01 and *** represent *p-value* < 0.001. The sums of the proportions of Spatial, Environmental and Residual do not always exactly add to 1. If the sum is less than 1, there was another proportion explained by the combination of Spatial and Environmental. If the sum is greater than 1, the proportion explained by the explanatory variables (Spatial, Environmental or the combination of Spatial and Environmental) was negative. A negative value indicates that the proportion explained by the explanatory variables was less than the random value (Borcard et al., 2019; Legendre & Legendre., 2012).  Borcard, D., Gillet, F., & Legendre, P. (2019). *Numerical Ecology with R, 2nd edition. Chinses edition* (translation: J. Lai, Institute of Botany, Chinese Academy of Sciences). Higher Education Press, Beijing.  Legendre, P., & Legendre, L. (2012). *Numerical ecology, 3rd English edition.* Elsevier Science BV. Amsterdam. | | | | | | | | | | |
